# Effects of an Empowerment-Based Health-Promotion School Intervention on Physical Activity and Sedentary Time among Adolescents in a Multicultural Area

**DOI:** 10.3390/ijerph15112542

**Published:** 2018-11-13

**Authors:** Andreas Fröberg, Linus Jonsson, Christina Berg, Eva-Carin Lindgren, Peter Korp, Magnus Lindwall, Anders Raustorp, Christel Larsson

**Affiliations:** 1 Department of Food and Nutrition, and Sport Science, University of Gothenburg, 41120 Gothenburg, Sweden; linus.jonsson@gu.se (L.J.); christina.berg@ped.gu.se (C.B.); eva-carin.lindgren@hh.se (E.-C.L.); peter.korp@ped.gu.se (P.K.); anders.raustorp@gu.se (A.R.); christel.larsson@gu.se (C.L.); 2 School of Health and Welfare, Halmstad University, 30118 Halmstad, Sweden; 3 Department of Psychology, University of Gothenburg, 40530 Gothenburg, Sweden; magnus.lindwall@gu.se; 4 Department of Sport Science, Linnaeus University, 391 82 Kalmar, Sweden

**Keywords:** empowerment, exercise, health promotion, participatory, physical activity, school, sedentary behavior

## Abstract

Physical activity (PA) decreases with age, and interventions are needed to promote PA during adolescence, especially, among those in low-socioeconomic status (SES) areas. The aim of this study was to investigate whether a two-year, empowerment-based health-promotion school intervention had any effects on changes in (a) moderate-to-vigorous PA (MVPA), (b) sedentary time (SED), (c) exercise training (ET) frequency, and (d) ET duration, among adolescents. Participants (aged 12–13 years at baseline) from one intervention school and two control schools, were recruited from a multicultural area of Sweden, characterized by low-SES. During the course of the two-year intervention, a total of 135 participants (43% boys) were included in the study. The intervention was developed and implemented as a result of cooperation and shared decision-making among the researchers and the participants. MVPA and SED were measured with accelerometers, and ET frequency and duration was self-reported at the beginning of the seventh, eighth, and ninth grade, respectively. There were no significant effects of the two-year, empowerment-based health-promotion school intervention on changes in the accelerometer-measured MVPA and SED, or the self-reported ET frequency and duration, among the adolescents. Overall, the intervention was unsuccessful at promoting PA and reducing SED. Several possible explanations for the intervention’s lack of effects are discussed.

## 1. Introduction

A body of evidence indicates that physical activity (PA), particularly at moderate and vigorous intensity (MVPA), is associated with wide-ranging health benefits, during childhood and adolescence [1,2]. It appears, however, that most adolescents in Western societies are not physically active enough to comply with the current PA recommendations [3]. Furthermore, studies suggest that PA decreases [4] and sedentary time [5] increases, during adolescence. Therefore, interventions are needed to promote PA, during adolescence. A number of interventions targeting adolescents have been implemented during recent decades, but they appear to have had somewhat limited success in promoting PA [6]. A recent meta-synthesis including thirteen interventions reported a non-significant effect on PA, among adolescents [6]. Currently, however, it appears less common to involve adolescents in the development and implementation of PA interventions. To give adolescents a voice and involve them in the identification of their health issues, development of appropriate solutions have recently been stressed upon [7]. Adolescents might possess unique perspectives on health habits that could be advantageously incorporated into interventions, to improve strategies, in terms of acceptability and appropriateness. Verloigne and co-workers [8] invited adolescent girls to use a co-creation process to develop a PA intervention. The co-creational intervention involved PAs, such as organized sports activities and fitness-inspired activities. Although no positive effects on PA were identified, the participants reported positive experiences regarding having a voice in developing the PA intervention [8]. In a study by Okely et al. [9], fourteen-year-old girls formed committees that were supported by researchers, to develop action plans for promoting PA. Similar to the study described above, no positive effects on PA were observed, but the authors concluded that the lack of implementation by most schools could explain the null effect [9].

Furthermore, the need for interventions to promote PA and to develop and implement interventions where adolescents are given the opportunity to be heard in matters affecting their health, might be especially important among youth from low-socioeconomic circumstances. Although debated, some research suggests a relationship between low-SES and lesser PA, among adolescents [10]. Recent trends also suggest that inequalities between socioeconomic groups have increased, with regards to PA among adolescents, during the last decade [11]. To support adolescents from low-SES areas to achieve and maintain healthy PA habits is, therefore, a priority.

The aim of the present study was to investigate whether a two-year, empowerment-based, health-promotion school intervention had any effects on changes in (a) accelerometer-measured MVPA, (b) accelerometer-measured sedentary time, (c) self-reported exercise training (ET) frequency, and (d) self-reported ET duration, among adolescents in a Swedish multicultural area characterized by low-SES.

## 2. Materials and Methods

This two-year intervention study involved three measurement points. Data for accelerometer-measured MVPA and sedentary time, self-reported ET frequency, and ET duration were collected in 2014 (T1, baseline), 2015 (T2, midpoint), and 2016 (T3, endpoint), in September of each year, when participants were in the seventh grade (ages 12–13 years), eighth grade (ages 13–14 years), and ninth grade (ages 14–15 years), respectively. T3 was conducted four months after the last intervention activity was held.

### 2.1. Participants

The study participants were recruited from three municipal schools (n = 1 intervention school and n = 2 control schools) in Angered of Gothenburg, Sweden. Angered is a multicultural area characterized by low-SES, as a high proportion (72%) of residents have foreign backgrounds; in addition, economic income and educational level are low, compared to the overall population living in the municipality of Gothenburg [12].

At a formal meeting in 2013, principals and representatives of all schools in the area of Angered (i.e., twenty schools) were presented with a proposal for the planned empowerment-based, health-promotion school intervention (the ‘How-to-Act?’ project), and the researchers expressed their wish to recruit one school in which to intervene. Thus, all schools were approached and asked to show their interest in participating. In total three schools showed interest in participating in the intervention. In order to practically perform the intervention only one school was planned to be recruited and in further dialog with the three schools the one which showed the most interest in participating, was recruited. Subsequently, two schools in the same area with comparable data for SES and educational achievement scores, which were low, compared to national standards, were recruited as control schools. Descriptive data (at T1) for the three schools involved in the study are presented in Table 1.

All one hundred and fifty-two pupils in the seventh grade, who attended the three schools at T1, were invited to participate in the study, as were the new pupils who transferred to any of the three schools, during the study period, except at T3. For pupils at the intervention school, this meant being involved in the intervention and taking part in the measurements. For pupils at the control school, this only involved the measurements. The pupils and their parents or legal guardians received written (in Swedish, Arabic, or Somali) and oral information, and all were given an opportunity to ask any questions they had about the study. It was clarified that participation was voluntary and that any participant could withdraw from the study, at any time, without providing any further explanation or justification.

A total of one hundred and thirty-five participants took part in the study during the two years. During T1, T2, and T3, the numbers were 114 (n = 54 intervention group), 110 (n = 53 intervention group), and 101 (n = 54 intervention group) participants involved in the intervention, respectively. The overall loss of participants from baseline to follow-up was 29%. In the intervention group, loss of participants from baseline to follow-up was 13%. A total of forty-six participants were involved during T1, T2, and T3; seven participants were involved during only two measurement points, and eight during only one measurement point (either T1, T2, or T3). In the control group, loss of participants from baseline to follow-up was 43%. A total of twenty-four participants were involved during T1, T2, and T3; twenty-four participants were involved during only two measurement points, and twenty-six during only one measurement point (either T1, T2, or T3).

The research conducted in this paper was reviewed and approved by an ethics committee (registration # 2014/469-14), and all participants and their parents or legal guardians provided signed written and informed consent, prior to their participation.

### 2.2. Intervention Aim and Design

The intervention was participatory-based and was supported by the idea of empowerment, thus, it followed an empowerment approach. The aim of the intervention was to explore how to support adolescents to achieve and maintain healthy food and PA habits.

The theoretical basis for the intervention was empowerment, which were referred to as possibilities for one to formulate and influence opportunities and barriers for change, and procuring motivation and belief in one’s own ability. The intervention embraced the ideas of empowerment as both a goal and a process [13]. As a goal, the intervention aimed to facilitate self-control, knowledge, autonomy, self-esteem, and self-confidence [13]. As a process, the aim was for the intervention to be continually developed and implemented, as a result of cooperation and shared decision making, where a central idea was that the researchers would support participants in expressing their goals, wishes, and needs; listen to their ideas; and put these suggestions into practice [13].

The intervention also relates to the ideas of the reflective equilibrium community-empowerment approach, which combines bottom-up and top-down approaches to include participants in the decision-making processes, while simultaneously acknowledging the need for the intervention to be guided by health information [14]. The reflective equilibrium community-empowerment approach also involves a process of dialogue between the researchers and the participants involved in an intervention [14]. In this regard, researchers should be responsive and willing to modify their views regarding effective strategies and which health-related issues to prioritize during the intervention [14]. The researchers used what they called a reflective spiral of cycles to guide the collaborative process of reflecting upon their experiences, participating in the intervention, and listening to participants’ comments, in search of possible actions for implementation. With the support and guidance of protocols through which the field-workers had documented expectations, experiences, and general observations, as well as the reflections made during the intervention, the researchers continuously reviewed and reflected upon these experiences (i.e., perceived barriers and opportunities) and ways to modify the intervention, to meet the participants’ goals, preferences, and needs. The researchers who participated in this reflection procedure formed a heterogeneous constellation of collaborators, who collectively possessed a broad interdisciplinary base of knowledge, in the fields of food and nutrition, sports science, and health promotion.

The intervention was developed and implemented, as a result of cooperation and shared decision-making among the researchers and the participants. To aid participation and support a sense of empowerment, the intervention relied on health coaching, health-promotion sessions, and a closed-Facebook-group.

Health coaching was used to involve participants in the development and implementation of the intervention, by supporting them in expressing their goals, wishes, and needs; listening to their suggestions about meeting them, and putting their suggestions into practice by developing and implementing intervention activities. These intervention activities consisted of health-promotion sessions, and the objective was that the theme, aim, and content of each health-promotion session would reflect not only the participants’ goals, wishes, and needs, but also the researchers’ common experiences and reasonable actions for implementation. The latter meant that the researchers reflected upon whether the participants’ suggestions about activities, during the health-promotion sessions (e.g., theme and content), could be delivered within the given time-frame and in the physical environment. It further meant that the researchers acknowledged the need for health information, as the goal was that the activities would provide opportunities for an engaging experience of healthy foods and PA.

Moreover, to provide a forum for communication among the researchers and the participants, a Facebook group was launched at the beginning of the intervention. Here the researchers posted information on upcoming health-promotion sessions, provided feedback, and wrote comments; participants could ask questions about the intervention and write their feedback and comments, on the posts.

A total of thirty-one health-promotion sessions with a focus on food and PA were developed and implemented, across the intervention. These sessions were conducted during school hours (n = 15 sessions, lasting 90 min; n = 13 sessions, lasting 60 min; and n = 3 sessions, lasting 120–180 min), and most were held in the school environment and its surroundings (classroom, gym, etc.), while some took place at other locations in the Gothenburg area. The average attendance rate among participants at the health-promotion sessions was 83–87%, depending on the semester. Examples of activities related to PA were brisk walking, using pedometers, performing body-weight-based resistance-training exercises, jogging/running, swimming, dancing, playing sports (e.g., soccer, basketball, martial arts), and the online searching and compiling of health benefits related to PA. Two PhD students (A.F. and L.J.) had the main responsibility of conducting the health coaching sessions, as well as implementing the health-promotion sessions. They were occasionally supported by other members of the research group, for example, during the whole day sessions. The design of the intervention was similar to what Salazar et al. [15] describe as a quasi-experimental, non-randomized, longitudinal pretest-posttest design. For further information about the intervention design, its development, and implementation see Jonsson et al [16]. Although the intervention addressed both PA and food habits, for the purpose of this paper, we only evaluated the effects of the intervention on PA and sedentary time. The effects of the intervention on food habits will be evaluated in another paper.

### 2.3. Measures

#### 2.3.1. Accelerometer

The number of minutes engaged in MVPA and sedentary time, each day, were measured with hip-mounted accelerometers (model GT3X/GT3X+, ActiGraph™ LCC, Pensacola, FL, USA). Prior to T1, T2, and T3, the participants were given oral and written instructions, as well as practical demonstrations about how to wear the accelerometer, appropriately. They were asked to wear the device during all waking hours, across one week, except when engaging in water-based activities (e.g., swimming, bathing, showering). Due to a limited number of available accelerometers, participants from the intervention group and the control group were measured during two different weeks (in between one-to-two weeks), at all three measurement points.

The data collected from the accelerometers at all three measurement points, were integrated into five-second durations to capture the adolescents’ intermittent and spontaneous PA behaviors [17]. Wearing time was defined by subtracting all sequences ≥60 consecutive minutes of 0 counts, from a 24-h monitoring period [18,19]. Participants were required to provide ≥3 days with ≥8 h per day of monitoring, to be included in the analysis. Cut-points, according to Evenson and co-workers [20] were used to estimate minutes per day of MVPA (≥2296 counts per minutes (CPM)) and sedentary time (≤100 CPM), as previously recommended [21].

#### 2.3.2. Questionnaire

ET frequency and ET duration were self-reported by two questions obtained from the Health Behavior in School-Aged Children (HBSC) study [22]. The two questions have been classified as having fair validity [23] and good reliability [23,24]. The underlying reason for including self-reported measures of ET was that accelerometers might be impractical to wear during some types of organized sports [25]. The questionnaire was completed in a home-classroom, during T1, T2, and T3, and researchers were present during the procedure, to provide information and answer any questions.

The following questions for ET frequency were formulated: (1) “Outside school hours, how often do you usually exercise in your free time so much that you get out of breath or sweat?” Seven possible options ranged from “never” to “every day.” In the analysis (see below), the seven possible responses for ET frequency were coded as follows: 1 = never; 2 = less than once a month; 3 = once a month; 4 = once a week; 5 = 2–3 times a week; 6 = 4–6 times a week; and 7 = every day. (2) ET duration was measured with the question: “Outside school hours, how many hours a week do you usually exercise in your free time so much that you get out of breath or sweat?” Six possible options ranged from “none” to “about 7 h or more.” In the analysis (see below), the six possible responses for ET duration were coded as follows: 1 = none; 2 = about 30 min; 3 = about 1 h; 4 = about 2–3 h; 5 = about 4–6 h; and 6 = about 7 h or more.

#### 2.3.3. Anthropometrics

Anthropometrics were collected during T1 and T3. Body weight (0.1 kg) and height (0.1 cm) were measured with electronic portable weight scales (Beurer GS 27, CE Utrecht, The Netherlands) and portable stadiometers (Seca 217, Birmingham, UK), respectively. Body mass index (BMI) was calculated and classified, according to sex and age-specific cut-offs [26].

#### 2.3.4. Statistics

Descriptive statistics were calculated using SPSS 24.0. To analyze changes in the different activity measures, latent growth curve analyses (LGCA) were used. LGCA enables opportunities to investigate both inter- and intra-individual changes over time. These analyses were performed using the Bayesian estimator in Mplus 8.0 [27].

With regards to the accelerometer-measured MVPA and sedentary time, in the first step (Model 1), an intercept (i.e., starting point) and a slope (i.e., change) from T1–T3 were specified, in the LGCA. In the second step (Model 2), the average accelerometer wearing time was included as a predictor for both the intercept and the slope. In the third step (Model 3), the intervention condition (i.e., intervention group vs. control group) was added as a predictor of both the intercept and the slope. Finally, in the fourth step (Model 4), sex was included as a predictor of both the intercept and the slope.

Regarding self-reported ET frequency and ET duration, in the first step (Model 1), an intercept (i.e., starting point) and a slope (i.e., change) from T1–T3 was specified in the LGCA. In the second step (Model 2), the intervention condition was included as a predictor of both the intercept and the slope. In the third step (Model 3), sex was added a predictor of both the intercept and the slope.

To handle missing data, for accelerometer-measured MVPA and sedentary time, and self-reported ET frequency and ET duration, we used the full information maximum likelihood (FIML) estimation. The FIML estimation has been suggested to be superior to more traditional missing data procedures (e.g., imputation, list wise deletion; for more information about the biases related to these procedures see, for example, Baraldi & Enders [28]). In this procedure all available data were used to identify the parameter values that had the highest probability of producing the sample data. Sensitivity analyses were performed to compare the results between the following models: (a) Intention to treat (all participants who had data from at least one measurement wave available), (b) only participants who had at data from at least two measurement waves were included, and (c) only participants who had data from all three waves were included. As all three different models showed similar results we decided to present the results from the intention to treat model, where the data from most participants could be included. Model fit was evaluated using the posterior predictive p (PPp) value, along with the 95% confidence interval. A model with an excellent fit was expected to have a PPp value around 0.5, in combination with a symmetric 95% confidence interval, centering on zero. For each parameter in the model, the credibility interval (CI) was calculated. The CI indicated the probability that the parameter lies between two values, given the observed data, and in cases where the 95% CI did not involve zero, the parameter estimate was considered credible (i.e., the null hypothesis was rejected as improbable). To evaluate which model demonstrated the best fit to the data, model comparisons were evaluated with the deviance information criterion (DIC), where a lower DIC value indicated a model with better fit [29].

## 3. Results

Table 2 presents descriptive data for the participants in the intervention group and the control group, respectively, during T1 and T3.

### 3.1. Changes in MVPA and Sedentary Time between T1 and T3

In total, accelerometer data were provided by sixty-five participants at T1 and T3 (n = 49 for all three measurement points), eighteen participants at T1 + T2 or T2 + T3, and the remaining thirty-nine at only one measurement point (Figure S1) (loss to follow-up: 45%). Mean daily accelerometer wearing times were 837 min (intervention group: 852 min; and control group: 822 min), 842 min (intervention group: 873 min; and control group: 800 min), and 828 min (intervention group: 839 min; and control group: 813 min) for T1, T2, and T3 respectively.

**Figure 1:**
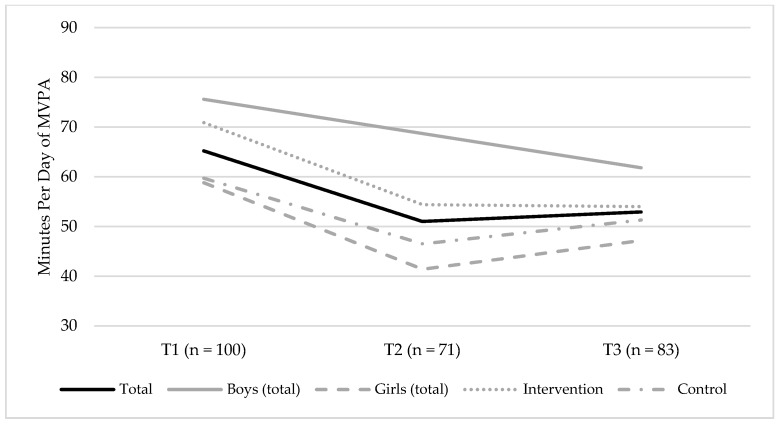
Changes in minutes per day of the accelerometer-measured moderate-to-vigorous physical activity (MVPA), between T1 (baseline, 2014) and T3 (endpoint, 2016), for the total sample, boys (total sample), and girls (total sample), and the intervention and control groups, respectively. The figure shows the mean group level of MVPA.

Changes in the accelerometer-measured MVPA between T1 and T3 are illustrated in Figure 1. There was a credible change in MVPA, which decreased by, approximately, 6.6 min per day (−6.58 [95% CI = −8.64; −4.49]) and year, equaling to, approximately, 13 min less per day of MVPA, during the two-year follow-up (Table S1). Model 4 shows the best fit to data. This model indicated that there was no credible effect of sex on the slope (β = −0.01, 95% CI = [−0.37; 0.34]), meaning that changes in MVPA were similar among boys and girls. Moreover, the model demonstrated that the intervention group had a higher level of MVPA at T1 (β = −0.20, 95% CI = [−0.37; −0.01]), but there was no credible effect of the intervention condition on the slope (β = 0.18, 95% CI = [−0.18; 0.56]), meaning that changes in MVPA were similar among participants in the intervention group and the control groups, respectively.

**Figure 2:**
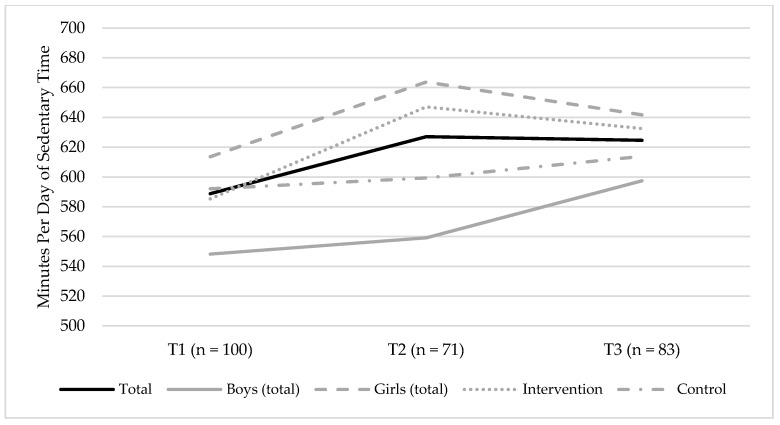
Changes in minutes per day of the accelerometer-measured sedentary time between T1 (baseline, 2014) and T3 (endpoint, 2016) for the total sample, boys (total sample), and girls (total sample), and the intervention and control groups, respectively. The figures show the mean group levels of sedentary time.

Changes in the accelerometer-measured sedentary time between T1 and T3 are illustrated in Figure 2. There was a credible change in sedentary time, which increased by 17.5 min per day (17.5 [95% CI = 0.81; 34.00]) and year, equivalent to 35 min more per day of sedentary time, during the two-year follow-up (Table S2). Model 4 demonstrated the best fit to data and showed that there was no credible effect of sex on the slope (β = 0.13, 95% CI = [−0.19; 0.46]), meaning that changes in sedentary time were equal among boys and girls. The model also demonstrated that the intervention group had a lower level of sedentary time at T1 (β = 0.26, 95% CI = [0.08; 0.43]), but there was no credible effect of the intervention condition on the slope (β = −0.19, 95% CI = [−0.55; 0.15]), meaning that changes in sedentary time were similar among participants in the intervention and control groups, respectively.

### 3.2. Changes in Exercise Training Frequency and Duration, between T1 and T3

A total of eighty-one participants provided data for the self-reported ET frequency, at T1 and T3 (n = 69 for all three measurement points), eighteen participants provided data at T1+T2 or T2+T3, and the remaining thirty-two participants only provided data for one measurement point (Figure S2) (loss to follow-up: 29%). The corresponding figures for the ET duration were as follows—seventy-nine participants provided data at T1 and T3 (n = 69 for all three measurement points), eighteen participants at T1 + T2 or T2 + T3, and the remaining thirty-one participants for one measurement point (Figure S3) (loss to follow-up: 27%).

**Figure 3:**
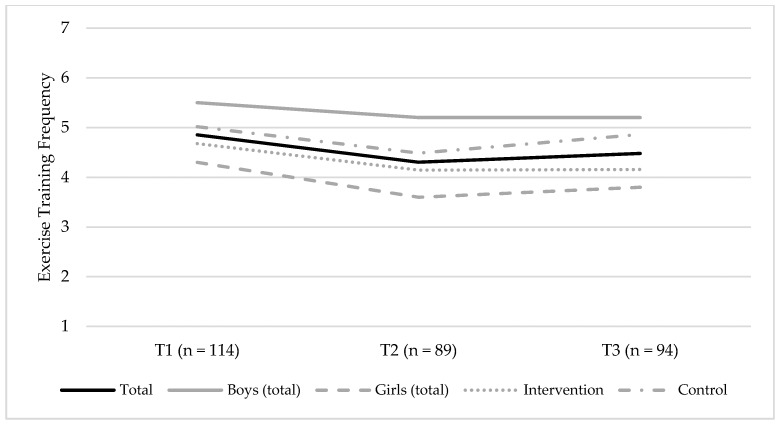
Changes in the self-reported exercise training (ET) frequency between T1 (baseline, 2014) and T3 (endpoint, 2016) for the total sample, boys (total sample) and girls (total sample), and the intervention group and control groups, respectively. The figure shows the mean group levels of ET frequency, and possible responses were coded as follows: 1 = never; 2 = less than once a month; 3 = once a month; 4 = once a week; 5 = 2–3 times a week; 6 = 4–6 times a week; and 7 = every day.

Changes in the self-reported ET frequency, between T1 and T3, are shown in Figure 3. There were no credible changes observed for the self-reported ET frequency (β = −0.20 [95% CI = −0.43; 0.20]) (Table S3). Model 3 showed the best fit to data and indicated no credible effect of sex on the slope (β = 0.01, 95% CI = [−0.27; 0.30]), meaning that changes in the ET frequency were equal among boys and girls. The model further demonstrated no credible effect of the intervention condition on the slope (β = 0.03, 95% CI = [−0.25; 0.33]), meaning that changes in the ET frequency were similar among participants in the intervention and control groups, respectively.

**Figure 4:**
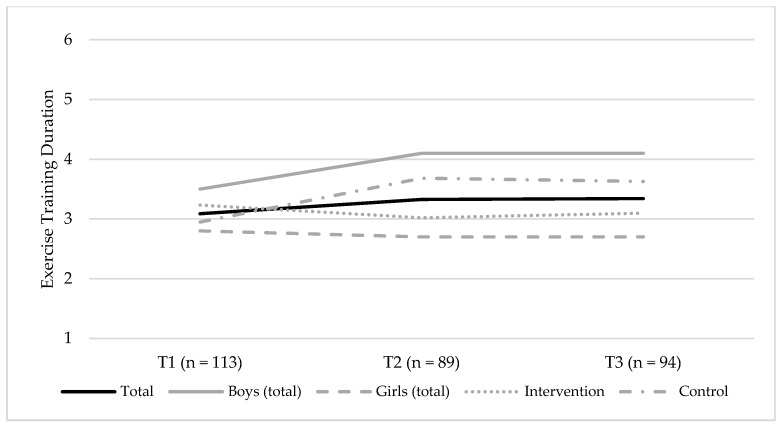
Changes in the self-reported exercise training (ET) duration, between T1 (baseline, 2014) and T3 (endpoint, 2016), for the total sample, boys (total sample), and girls (total sample), and the intervention and control groups, respectively. The figure shows the mean group levels of ET duration, and the possible responses were coded as follows: 1 = none; 2 = about 30 min; 3 = about 1 h; 4 = about 2–3 h; 5 = about 4–6 h; and 6 = about 7 h or more.

Changes in the self-reported ET duration, between T1 and T3 are shown in Figure 4. There were no credible changes observed for the self-reported ET duration (β = 0.14 [95% CI = −0.04; 0.34]) (Table S4). Model 3 showed the best fit to data. In this model, no credible effect of sex was observed on the slope (β = 0.24, 95% CI = [−0.02; 0.56]), meaning that changes in ET duration were equal among boys and girls. The intervention condition was a credible predictor of slope (β = 0.27 [95% CI = 0.01; 0.60]) where the control group, in comparison to the intervention group, had a more positive trajectory in the ET duration, between T1 and T3.

## 4. Discussion

There were no significant intervention effects on the accelerometer-measured MVPA and sedentary time or on the self-reported ET frequency or ET duration. These results for the accelerometer-measured MVPA are similar to those presented in a recent systematic review and a meta-analysis that showed that PA interventions, on average, have been unsuccessful in promoting MVPA among adolescents [6]. The results also align with those from some previous participatory-based PA interventions that show no effects on PA [8,9].

Reflecting with self-criticism, the researchers realize that the intervention featured several characteristics that, collectively, engendered a number of challenges. The intervention involved a complex process, as it was continually developed and implemented as a result of cooperation and shared decision-making among the researchers and participants, and much of the intervention’s success was due to the extent that structured group health-coaching sessions worked according to intentions. However, this complex health-coaching process was continually challenged, as participants tended to interrupt others and expressed disappointment with discussing goals related to food and PA habits [16]. Furthermore, participants were adolescents attending seventh grade at the onset of the intervention, and adolescence is recognized to be a turbulent stage of life [7]. The researchers anticipated that it would be challenging, but not impossible, to work with the goal-setting strategies, during our intervention; nonetheless, they experienced that participants seemed to live for and act in the present and had limited interest in formulating goals and working with goal-setting strategies [16]. The adolescents’ tendency to live and act for the present is perhaps not surprising, since adolescents, compared to adults, seem to lack a future orientation [30]. The researchers also experienced barriers, in relation to the context of the intervention, as it was partly chaotic, which, accordingly, contributed to occasional inattention among participants, during both the structured group health-coaching sessions and the health-promotion sessions [16].

Despite the barriers encountered during the two years of intervention, thirty-one health-promotion sessions, including a wide range of PAs (e.g., brisk walking with pedometers, jogging/running, swimming, dancing, and playing sports) were developed and implemented, as a result of the cooperation and shared decision-making among the researchers and participants. Taking into consideration that these thirty-one health-promotion sessions were spread over three school semesters, it is possible that the intervention’s duration and intensity were insufficient to bring about a change in PA. In addition, not all health-promotion sessions featured actual PAs, as some involved, for example, online searches and compiling health benefits related to PA, and food-related activities. Moreover, the intervention was individual and group centered, in the sense that it focused on the participants’ goals, wishes, and needs, in relation to food and PA, and most health-promotion sessions were conducted in the school environment. It is possible that the intervention would have benefited from an involvement of the family members in cooperating and developing and/or implementing the strategies to support PA, in the home environment, and during leisure time. Family-based intervention strategies, such as combinations of goal-setting and reinforcement techniques, in conjunction with interventions focusing on the benefits of spending time being physically active as a family, have been shown to be effective in increasing PA among the youth [31]. Furthermore, including family members in the intervention might have been essential, since, for example, focus-group interviews have revealed that participating girls express that their parents have strong expectations of them with regards to benefiting the household and family by doing chores (e.g., cooking and cleaning, caring for siblings), which they perceived to restrict their leisure-time PA [32]. Thus, involving family members in the intervention through workshops emphasizing the importance of being physically active and outlining strategies to spend time being physically active as a family, could have been a fruitful way to promote PA among the participants. However, the experiences of the homeroom teachers suggested a number of challenges (e.g., language barriers, lack of interest, and lack of time; some parents would be interested but have no time to become involved) regarding engaging the participants’ family members, in projects similar to the ‘How-to-Act?’ project.

Another possible explanation is that the socioeconomic conditions that characterize the participants’ everyday lives are significantly more important and affect them to a larger extent than any effects that school interventions, similar to the one reported here, might bring about. In this sense, systematic overviews focusing solely on interventions among youth from low-SES circumstances, overall, report limited success in promoting PA [33,34,35]. It has been suggested that intensive interventions targeting not only individual level, but also the family, the school, and the societal levels, are needed for youths to adopt healthy lifestyles [36].

Moreover, T3 was conducted approximately four months after the last intervention activity was held. A previous meta-analysis involving thirty interventions (n = 17 were school-based trials) with the accelerometer data measured, either before or immediately after the end of the intervention, found an average of four additional minutes of the accelerometer-measured MVPA, among youth [37]. However, in another meta-analysis involving studies with a follow-up measurement data at least six months post-intervention, found a non-significant effect on MVPA [38]. Thus, perhaps an intervention effect would have been detected if T3 had been conducted, either before or immediately after the end of the intervention; yet, in the longer term, the effect might have disappeared.

It should further be noted that, due to restrictions in the number of accelerometers available, the participants at the intervention school and the two control schools were measured during two different weeks with one to two week(s) in between. Data obtained from the Swedish Meteorological and Hydrological Institute (SMHI) showed that the amount of precipitation was higher during the week the intervention group was measured, at both T2 and T3. In particular, the amount of precipitation was higher at T3 with 20.6 mm for the intervention group and 0.0 mm and 6.1 mm for the two control schools, respectively. Previous research indicates that higher amounts of precipitation might be associated with less PA [39] and these differences in weather conditions might have influenced the results. Moreover, during the T3 fieldwork, participants in the intervention group explicitly mentioned the amount of precipitation as a reason for engaging in less PA.

Although no effects on the accelerometer-measured MVPA and sedentary time were observed, it is further acknowledged that the activities performed during the intervention, featured elements and qualities that the participants themselves perceived as being positive in relation to their PA habits. Qualitative data from the focus-group interviews conducted among the participants in the intervention group, as part of T3, suggested that they felt that they were listened to, throughout the intervention, and had been given opportunities to influence and decide on the content related to PA, during the health-promotion sessions [40]. They also stated that they had realized that PA could be enjoyable and had perceived themselves as gaining more PA skills, as well as having become more aware of their PA habits [40]. Such positive experiences of participating in the intervention might not necessarily have had any effects on the accelerometer-measured MVPA and sedentary time, but they may have been viewed as prerequisites to becoming more physically active in the longer term.

For the total sample, this study showed an annual decrease in the accelerometer-measured MVPA, of seven minutes per day (i.e., 10% per day annually), an annual increase in the accelerometer-measured sedentary time, of eighteen minutes per day (i.e., 3% per day and year), and no changes for the self-reported ET frequency or ET duration. The decrease in MVPA seems to be somewhat larger than in previous studies [41,42,43], while the increase in sedentary time appears to be somewhat smaller [41,42,43].

Similar to other studies, the results presented here highlight the need for effective interventions to promote PA during adolescence. It should be noted, however, that findings from the International Children’s Accelerometry Database (ICAD) suggest that PA begins to decrease during school entry and continues throughout adolescence [3]. This is further supported by a recent longitudinal studies measuring PA by accelerometry, which show that PA gradually decreases with age [44]. Thus, effective interventions to promote PA should reasonably begin at early ages. However, as data further show that PA generally decreases from the transition of adolescence to early adulthood [45], interventions to promote PA, during adolescence, to counteract further decrease during adulthood, might have important implications.

The strengths of this study include the use of accelerometers to measure the effects of the two-year, empowerment-based, health-promotion school intervention on MVPA and sedentary time, among adolescents from multicultural areas, characterized by low-SES. Limitations of this study include the recruitment procedure and the lack of randomization to intervention/control condition, the small sample size (only one intervention school and one hundred and thirty-five participants included during the two years of intervention), loss of participants from baseline to follow-up, and the fact that relatively few participants provided sufficient accelerometer data for all three measurement points. Further, many previous accelerometer-based studies have required ≥3–4 days with ≥ 8–10 h per day of monitoring to be included in the analysis [46]. To maximize the sample size in this study, ≥3 days with ≥8 h per day of monitoring, was the wear-time criterion. However, a higher wear-time criterion would likely have increased the reliability of the MVPA and sedentary time estimates and this is acknowledged as a limitation. Further, as adolescents generally engage in more PA during weekdays, as compared to the weekend [47], another limitation is the lack of requirements of including ≥1 weekend day(s), within the wear-time criterion.

## 5. Conclusions

There were no significant effects of a two-year, empowerment-based, health-promotion school intervention on the changes in accelerometer-measured MVPA and sedentary time, or the self-reported ET frequency and ET duration, among adolescents in a Swedish multicultural area, characterized by low-SES. In the whole sample, MVPA decreased and sedentary time increased annually by 7 mins and 18 mins per day, respectively. No changes were observed for either the ET frequency or the ET duration, over time. To prevent a daily decrease in MVPA and increase in the sedentary time, among adolescents in a Swedish multicultural area, characterized by low-SES, a more intensive intervention targeting not only individual levels at school, but also at the family and societal levels, seems to be needed. However, although no effects on the accelerometer-measured MVPA and sedentary time were observed, the activities performed during the intervention were perceived as being positive experiences in relation to PA habits, which may be prerequisites to becoming more physically active, in the longer term.

## Figures and Tables

**Table 1:** Descriptive data for the three schools involved in the intervention at T1 (2014).

	Intervention	Control 1	Control 2	National
Pupil–teacher ratio ^1^	10.3	11.5	8.0	12.1
Socioeconomic status ^2^	1.66	1.69	2.15	2.26
Educational achievement scores ^3^	185	162	212	225
Pass in all grades (%)	45.2	24.5	63.6	77.0
New arrivals ^4^ (%)	19	27	16	5
Foreign background ^5^ (%)	92	93	75	21

^1^ Number of pupils divided by number of teachers; ^2^ Mean of parents’ completed educational levels: 1 point = compulsory school, 2 points = upper-secondary school, 3 points = greater than or equal to twenty post-secondary education credits (Swedish National Agency for Education, available at www.siris.skolverket.se); ^3^ Scores of sixteen curriculum subjects ranging from 0 to 320 points: 20 points = grade A, 17.5 = B, 15 = C, 12.5 = D, 10 = E, 0 = F (The Swedish National Agency for Education, available at www.siris.skolverket.se); ^4^ Foreign-born pupils (with foreign-born parents) who arrived in Sweden during the previous four years, with no experience of the Swedish compulsory school system (Swedish National Agency for Education, available at www.siris.skolverket.se); ^5^ Foreign-born pupils and pupils born in Sweden with both parents born outside Sweden (Swedish National Agency for Education, available at www.siris.skolverket.se).

**Table 2:** Descriptive data for participants in the intervention group and the control group, during T1 (baseline, 2014) and T3 (endpoint, 2016), respectively.

								BMI Categories			
			n	Age, M years (±SD)	Height, M cm (±SD)	Weight, M kg (±SD)	BMI	Underweight, %	Normal Weight, %	Overweight, %	Obese, %
Total Sample	T1			n = 114 (n = 66 girls)	n = 106 (n = 61)	n = 106 (n = 61 girls)	n = 106 (n = 61 girls)	n = 10 (n = 6 girls)	n = 59 (n = 33 girls)	n = 26 (n = 13 girls)	n = 11 (n = 9 girls)
		Total	114	12.8 (±0.5)	160.0 (±7.4)	53.8 (±12.6)	20.9 (±4.4)	9.4	55.7	24.5	10.4
		Girls	66	12.8 (±0.4)	158.5 (±6.7)	54.6 (±13.3)	21.6 (±4.7)	9.8	54.1	21.3	14.8
		Boys	48	12.8 (±0.5)	162.0 (±8.0)	52.6 (±11.6)	20.0 (±3.9)	8.9	57.8	28.9	4.4
	T3			n = 98 (n = 55 girls)	n = 94 (n = 53 girls)	n = 93 (n = 51 girls)	n = 93 (n = 51 girls)	n = 5 (n = 2 girls)	n = 55 (n = 28 girls)	n = 22 (n = 15 girls)	n = 11 (n = 6 girls)
		Total	98	14.8 (±0.5)	166.3 (±8.5)	63.2 (±15.0)	22.8 (±4.8)	5.4	59.1	23.7	11.8
		Girls	55	14.8 (±0.4)	161.2 (±6.3)	60.9 (±13.7)	23.4 (±4.7)	3.9	54.9	29.4	11.7
		Boys	43	14.8 (±0.5)	172.5 (±6.5)	66.1 (±16.2)	22.1 (±4.9)	7.1	64.3	16.7	11.9
Intervention Group	T1			n = 54 (n = 32 girls)	n = 54 (n = 32 girls)	n = 54 (n = 32 girls)	n = 54 (n = 32 girls)	n = 5 (n = 3 girls)	n = 26 (n = 17 girls)	n = 16 (n = 7 girls)	n = 7 (n = 5 girls)
		Total	54	12.8 (±0.5)	159.7 (±7.6)	55.7 (±13.0)	21.7 (±4.4)	9.3	48.1	29.6	13.0
		Girls	32	12.8 (±0.4)	158.1 (±6.6)	55.1 (±12.9)	21.9 (±4.5)	9.4	53.1	21.9	15.6
		Boys	22	12.8 (±0.6)	161.9 (±8.5)	56.5 (±13.5)	21.4 (±4.4)	9.1	40.9	40.9	9.1
	T3			n = 51 (n = 31 girls)	n = 51 (n = 31 girls)	n = 51 (n = 31 girls)	n = 21 (n = 31 girls)	n = 3 (n = 1 girls)	n = 25 (n = 16 girls)	n = 14 (n = 10 girls)	n = 9 (n = 4 girls)
		Total	51	14.8 (±0.5)	165.3 (±8.5)	65.2 (±8.5)	23.7 (±5.1)	5.9	49.0	27.5	17.6
		Girls	31	14.8 (±0.4)	160.7 (±6.0)	61.0 (±13.3)	23.5 (±4.5)	3.2	51.6	32.3	12.9
		Boys	20	14.8 (±0.5)	172.5 (±6.7)	71.6 (±19.9)	23.9 (±6.0)	10.0	45.0	20.0	25.0
Control Group	T1			n = 60 (n = 34 girls)	n = 52 (n = 29 girls)	n = 52 (n = 29 girls)	n = 52 (n = 29 girls)	n = 5 (n = 3 girls)	n = 33 (n = 16 girls)	n = 10 (n = 6 girls)	n = 4 (n = 4 girls)
		Total	60	12.8 (±0.5)	160.4 (±7.3)	51.9 (±11.9)	20.1 (±8.3)	9.6	63.5	19.2	7.7
		Girls	34	12.8 (±0.4)	158.9 (±6.9)	54.2 (±13.9)	21.3 (±4.9)	10.3	55.2	20.7	13.8
		Boys	26	12.9 (±0.5)	162.1 (±7.6)	49.0 (±8.3)	18.6 (±2.8)	8.7	73.9	17.4	0.0
	T3			n = 47 (n = 24 girls)	n = 43 (n = 21 girls)	n = 42 (n = 20 girls)	n = 42 (n = 20 girls)	n = 2 (n = 1 girls)	n = 30 (n = 12 girls)	n = 8 (n = 5 girls)	n = 2 (n = 2 girls)
		Total	47	14.8 (±0.5)	167.4 (±8.4)	60.9 (±12.1)	21.8 (±4.1)	4.8	71.4	19.0	4.8
		Girls	24	14.8 (±0.4)	161.9 (±6.7)	60.8 (±14.5)	23.2 (±4.9)	5.0	60.0	25.0	10.0
		Boys	23	14.9 (±0.5)	172.6 (±6.5)	61.1 (±9.8)	20.5 (±2.7)	4.5	81.8	13.6	0.0

Abbreviations: BMI, Body Mass Index.

## Supplementary Materials

The following are available online at http://www.mdpi.com/1660-4601/15/11/2542/s1: Figure S1: Flowchart of accelerometer data; Figure S2: Flowchart of data for exercise training frequency; Figure S3: Flowchart of data for exercise training duration. Table S1. Models 1–4 for changes in MVPA, between T1 and T3; Table S2. Models 1–4 for changes in sedentary time, between T1 and T3; Table S3. Models 1–3 for changes in ET frequency, between T1 and T3; Table S4. Models 1–3 for changes in ET duration, between T1 and T3.
