# Supplementary material for: Effects of an Empowerment-Based Health-Promotion School Intervention on Physical Activity and Sedentary Time among Adolescents in a Multicultural Area
Source: Int J Environ Res Public Health. 2018 Nov 13;15(11):2542. doi: 10.3390/ijerph15112542 (PMC6267499; doi:10.3390/ijerph15112542)
Supplement: Supplementary file 1 [file ijerph-15-02542-s001.docx]

**Table S1**. **Model 1-4** for changes in moderate-to-vigorous physical activity (MVPA) between T1 and T3.

| **Parameters of the model** | **Model 1** | **Model 2** | **Model 3** | **Model 4** |
| --- | --- | --- | --- | --- |
| *Means* |  |  |  |  |
| Level of MVPA | 65.15 (60.83; 70.05) | NA | NA | NA |
| Change in MVPA | -6.58 (-8.64; -4.49) | NA | NA | NA |
|  |  |  |  |  |
| *Regression weights* |  |  |  |  |
| Wear-time – intercept |  | -0.09 (-0.28; 0.11) | -0.17 (-0.36; 0.04) | -0.08 (-0.27; 0.12) |
| Wear-time – slope |  | -0.26 (-0.64; 0.12) | -0.18 (-0.57; 0.20) | -0.22 (-0.60; 0.18) |
| Intervention vs control – intercept |  |  | -0.22 (-0.40; -0.02) | -0.20 (-0.37; -0.01) |
| Intervention vs control – slope |  |  | 0.19 (-0.16; 0.56) | 0.18 (-0.18; 0.56) |
| Sex – intercept |  |  |  | 0.34 (0.16; 0.50) |
| Sex - slope |  |  |  | -0.01 (-0.37; 0.34) |
|  |  |  |  |  |
| *Model fit* |  |  |  |  |
| DIC | 2235.44 | 2233.22 | 2223.20 | 2217.18 |
| PPP | 0.24 | 0.26 | 0.26 | 0.20 |

**Note**: Credible predictors indicated as bolded

*Model 1: No predictors added*

*Model 2: Added predictors: accelerometer wear-time*

*Model 3: Added predictors: accelerometer wear-time and intervention group-belonging*

*Model 4: Added predictors: accelerometer wear-time, intervention group-belonging, and sex*

*DIC: Deviance information criterion*

*PPp: Posterior predictive p*

**Table S2**. **Model 1-4** for changes in sedentary time between T1 and T3.

| **Parameters of the model** | **Model 1** | **Model 2** | **Model 3** | **Model 4** |
| --- | --- | --- | --- | --- |
| *Means* |  |  |  |  |
| Level of sedentary time | 588.50 (567.30; 609.80) | NA | NA | NA |
| Change in sedentary time | 17.50 (0.81; 34.00) | NA | NA | NA |
|  |  |  |  |  |
| *Regression weights* |  |  |  |  |
| Wear-time – intercept |  | 0.80 (0.58; 0.97) | 0.87 (0.67; 1.00) | 0.81 (0.61; 0.96) |
| Wear-time – slope |  | 0.59 (0.24; 0.91) | 0.47 (0.11; 0.81) | 0.51 (0.16; 0.84) |
| Intervention vs control – intercept |  |  | 0.29 (0.11; 0.46) | 0.26 (0.08; 0.43) |
| Intervention vs control - slope |  |  | -0.21 (-0.57; 0.13) | -0.19 (-0.55; 0.15) |
| Sex – intercept |  |  |  | -0.22 (-0.39; -0.04) |
| Sex - slope |  |  |  | 0.13 (-0.19; 0.46) |
|  |  |  |  |  |
| *Model fit* |  |  |  |  |
| DIC | 3153.75 | 3048.60 | 3042.50 | 3039.30 |
| PPp | 0.45 | 0.22 | 0.22 | 0.25 |

**Note**: Credible predictors indicated as bolded

*Model 1: No predictors added*

*Model 2: Added predictors: accelerometer wear-time*

*Model 3: Added predictors: accelerometer wear-time and intervention group-belonging*

*Model 4: Added predictors: accelerometer wear-time, intervention group-belonging, and sex*

*DIC: Deviance information criterion*

*PPp: Posterior predictive p*

**Table S3**. **Model 1-3** for changes in exercise training (ET) frequency between T1 and T3.

| **Parameters of the model** | **Model 1** | **Model 2** | **Model 3** |
| --- | --- | --- | --- |
| *Means* |  |  |  |
| Level of ET frequency | 4.81 (4.48; 5.14) | NA | NA |
| Change in ET frequency | -0.20 (-0.43; 0.20) | NA | NA |
|  |  |  |  |
| *Regression weights* |  |  |  |
| Intervention vs control – intercept |  | 0.11 (-0.10; 0.32) | 0.10 (-0.10; 0.30) |
| Intervention vs control – slope |  | 0.07 (-0.21; 0.35) | 0.03 (-0.25; 0.33) |
| Sex – intercept |  |  | 0.42 (0.22; 0.62) |
| Sex – slope |  |  | 0.01 (-0.27; 0.30) |
|  |  |  |  |
| *Model fit* |  |  |  |
| DIC | 1171.93 | 1172.00 | 1149.52 |
| PPP | 0.32 | 0.38 | 0.39 |

**Note**: Credible predictors indicated as bolded

*Model 1: No predictors added*

*Model 2: Added predictors: intervention group-belonging*

*Model 3: Added predictors: intervention group-belonging and sex*

*DIC: Deviance information criterion*

*PPp: Posterior predictive p*

**Table S4**. **Model 1-3** for changes in exercise training (ET) duration between T1 and T3.

| **Parameters of the model** | **Model 1** | **Model 2** | **Model 3** |
| --- | --- | --- | --- |
| *Means* |  |  |  |
| Level of ET duration | 3.10 (2.84-3.35) | NA | NA |
| Change in ET duration | 0.14 (-0.04; 0.34) | NA | NA |
|  |  |  |  |
| *Regression weights* |  |  |  |
| Intervention vs control – intercept |  | -0.10 (-0.30; 0.12) | -0.10 (-0.30; 0.12) |
| Intervention vs control – slope |  | 0.32 (0.06; 0.66) | 0.27 (0.01; 0.60) |
| Sex – intercept |  |  | 0.33 (0.11; 0.58) |
| Sex – slope |  |  | 0.24 (-0.02; 0.56) |
|  |  |  |  |
| *Model fit* |  |  |  |
| DIC | 1054.47 | 1052.01 | 1027.27 |
| PPP | 0.52 | 0.41 | 0.38 |

**Note**: Credible predictors indicated as bolded

*Model 1: No predictors added*

*Model 2: Added predictors: intervention group-belonging*

*Model 3: Added predictors: intervention group-belonging and sex*

*DIC: Deviance information criterion*

*PPp: Posterior predictive*
