# Supplementary material for: Effects of an Empowerment-Based Health-Promotion School Intervention on Physical Activity and Sedentary Time among Adolescents in a Multicultural Area
Source: Int J Environ Res Public Health. 2018 Nov 13;15(11):2542. doi: 10.3390/ijerph15112542 (PMC6267499; doi:10.3390/ijerph15112542)
Supplement: Supplementary file 2 [file ijerph-15-02542-s002.docx]

**Figure S1: Accelerometer-Data**

**Note**: **T1:** baseline, 2014; **T2:** midpoint, 2015; and **T3:** endpoint, 2016.

**Three measurement points**

T1+T2+T3: *n* = 49

**Three measurement points**

T1+T2+T3: *n* = 15

**Three measurement points**

T1+T2+T3: *n* = 34

**Two measurement points**

T1+T2: *n* = 69

T1+T3: *n* = 16

T2+T3: *n* = 12

**Two measurement points**

T1+T2: *n* = 4

T1+T3: *n* = 9

T2+T3: *n* = 8

**Two measurement points**

T1+T2: *n* = 2

T1+T3: *n* = 7

T2+T3: *n* = 4

**One measurement point**

T1: *n* = 29

T2: *n* = 49

T3: *n* = 69

**One measurement point**

T1: *n* = 23

T2: *n* = 32

T3: *n* = 32

**One measurement point**

T1: *n* = 6

T2: *n* = 1

T3: *n* = 3

**Valid accelerometer-data**

T1: *n* = 51

T2: *n* = 30

T3: *n* = 35

**Valid accelerometer-data**

T1: *n* = 100

T2: *n* = 712

T3: *n* = 832

**Valid accelerometer-data**

T1: *n =* 49

T2: *n* = 41

T3: *n* = 48

**Total Sample**

**Control Group**

**Intervention Group**

**Figure S2: Exercise Training Frequency**

**Note**: **T1:** baseline, 2014; **T2:** midpoint, 2015; and **T3:** endpoint, 2016.

**Three measurement points**

T1+T2+T3: *n* = 69

**Three measurement points**

T1+T2+T3: *n* = 24

**Three measurement points**

T1+T2+T3: *n* = 45

**Two measurement points**

T1+T2: *n* = 11

T1+T3: *n* = 12

T2+T3: *n* = 72

**Two measurement points**

T1+T2: *n* = 9

T1+T3: *n* = 9

T2+T3: *n* = 6

**Two measurement points**

T1+T2: *n* = 2

T1+T3: *n* = 3

T2+T3: *n* = 1

**One measurement point**

T1: *n* = 24

T2: *n* = 29

T3: *n* = 69

**One measurement point**

T1: *n* = 18

T2: *n* = 22

T3: *n* = 42

**One measurement point**

T1: *n* = 4

T2: *n* = 0

T3: *n* = 2

**Questionnaire-data**

T1: *n* = 60

T2: *n* = 41

T3: *n* = 43

**Questionnaire-data**

T1: *n* = 114

T2: *n* = 89s

T3: *n* = 94s

**Questionnaire-data**

T1: *n =* 54

T2: *n* = 48

T3: *n* = 51

**Total Sample**

**Control Group**

**Intervention Group**

**Figure S3: Exercise Training Duration**

**Intervention Group**

**Control Group**

**Total Sample**

**Questionnaire-data**

T1: *n =* 54

T2: *n* = 48

T3: *n* = 51

**Questionnaire-data**

T1: *n* = 113

T2: *n* = 89s

T3: *n* = 94s

**Questionnaire-data**

T1: *n* = 59

T2: *n* = 41

T3: *n* = 43

**One measurement point**

T1: *n* = 6

T2: *n* = 0

T3: *n* = 2

**One measurement point**

T1: *n* = 17

T2: *n* = 22

T3: *n* = 42

**One measurement point**

T1: *n* = 23

T2: *n* = 20

T3: *n* = 69

**Two measurement points**

T1+T2: *n* = 2

T1+T3: *n* = 3

T2+T3: *n* = 1

**Two measurement points**

T1+T2: *n* = 90

T1+T3: *n* = 10

T2+T3: *n* = 50

**Two measurement points**

T1+T2: *n* = 11

T1+T3: *n* = 13

T2+T3: *n* = 63

**Three measurement points**

T1+T2+T3: *n* = 45

**Three measurement points**

T1+T2+T3: *n* = 24

**Three measurement points**

T1+T2+T3: *n* = 69

**Note**: **T1:** baseline, 2014; **T2:** midpoint, 2015; and **T3:** endpoint, 2016.
